# Long non-coding RNA Rpph1 promotes inflammation and proliferation of mesangial cells in diabetic nephropathy via an interaction with Gal-3

**DOI:** 10.1038/s41419-019-1765-0

**Published:** 2019-07-08

**Authors:** Panyang Zhang, Yan Sun, Rui Peng, Wenyun Chen, Xia Fu, Luyu Zhang, Huimin Peng, Zheng Zhang

**Affiliations:** 1 Molecular Medicine and Cancer Research Center, Chongqing Medical University, 400016 Chongqing, China; 2 Department of Bioinformatics, Chongqing Medical University, 400016 Chongqing, China; 3 People’s Hospital of Fuling District, 408000 Chongqing, China

**Keywords:** Mechanisms of disease, Long non-coding RNAs

## Abstract

Diabetic nephropathy (DN) is one of the most significant complications of diabetes and is the primary cause of end-stage kidney disease. Cumulating evidence has shown that renal inflammation plays a role in the development and progression of DN, but the exact cellular mechanisms are unclear. Irregular expression of long non-coding RNAs (lncRNAs) is present in many diseases, including DN. However, the relationship between lncRNAs and inflammation in DN is unclear. In this study, we identified differentially expressed lncRNAs in DN using RNA-sequencing. Among these lncRNAs, we identified seven DN-related lncRNAs in vivo and in vitro using quantitative real-time PCR. One lncRNA in particular, Rpph1 (ribonuclease P RNA component H1), exhibited significantly increased expression. Further, over-expression or knockdown of Rpph1 was found to regulate cell proliferation and the expression of inflammatory cytokines in mesangial cells (MCs). The results revealed that Rpph1 directly interacts with the DN-related factor galectin-3 (Gal-3). Further, over-expression of Rpph1 promoted inflammation and cell proliferation through the Gal-3/Mek/Erk signaling pathway in MCs under low glucose conditions, while knockdown of Rpph1 inhibited inflammation and cell proliferation through the Gal-3/Mek/Erk pathway in MCs under high glucose conditions. These results provide new insight into the association between Rpph1 and the Gal-3/Mek/Erk signaling pathway during DN progression.

## Introduction

Diabetic nephropathy (DN) is one of the main causes of end-stage renal disease^1^. In reality, the pathogenesis of DN is diverse, involving many mechanisms and factors, including inflammation and oxidative stress^2–4^. Recent studies suggest that renal inflammation is essential for promoting DN development^5,6^. Many scholars have found that inflammation can lead to kidney damage in the development of DN^7–9^. Inflammation may be a pivotal factor in stimulating known biochemical and metabolic disorders in DN through multifarious inflammation-associated cytokines, including tumor necrosis factor-α (Tnf-α) and monocyte chemoattractant protein-1 (Mcp-1)^10,11^. Although inflammation has been considered as an important link in the process of DN, its specific mechanism remains unclear and the current diagnosis and treatment methods are not ideal.

Long non-coding RNA (lncRNAs) with a length >200nt have become a focus of concern; these nucleotides lack the ability to encode proteins^12^. LncRNAs can directly interact with several RNA molecules or proteins to regulate the expression of target genes at the transcriptional and post-transcriptional level^13–15^. They play a key role in many life processes, such as epigenetic regulation, cell cycle regulation, and differentiation regulation^16,17^. LncRNAs have been found to affect the inflammation in various diseases, including DN^18,19^. However, the exact role of lncRNAs in DN inflammation is unclear.

Mitogen-activated protein kinase (MAPK) is an important member of the Ser/Thr protein kinase family^20^. There are four MAPK pathways composed of four signaling families: the extracellular signal-regulated kinase (ERK), big MAP kinase-1, c-Jun N-terminal kinase, and p38 signaling families^21^. The MAPK/ERK pathway, also known as the Ras-Raf-MEK-ERK pathway, and MAPK kinases (MAPKKs) are downstream signal molecules in the Ras signaling pathway. MAPKKs comprise MAPK kinase-1 (Mek1) and MAPK kinase 2 (Mek2). Further downstream are extracellular signal-regulated kinase-1 (Erk1) and extracellular signal-regulated kinase 2 (Erk2)^22^. Activated Erk enters the nucleus, activates c-Fos, c-Jun, and other transcriptional regulatory factors, and finally, jointly activates specific gene expression^20,23,24^. In recent years, several studies have reported that the activation of the MAPK/ERK signaling pathway can regulate the release of pro-inflammatory chemokines such as Tnf-α and Mcp-1 in DN^25–27^. However, there remains a lack of detailed understanding of the mechanism underlying the association between the MAPK/ERK pathway and inflammation in DN.

To investigate the potential relationship between lncRNAs and inflammation in DN, in this study, we identified dysexpressed lncRNAs using RNA-sequencing (RNA-seq) and quantitative real-time PCR (qRT-PCR) in the renal tissue of db/db DN mice and in mesangial cells (MCs) cultured under high glucose conditions (H-MCs). LncRNA Ribonuclease P RNA component H1 (Rpph1) was found to be related to DN, and the expression of Rpph1 was most obviously changed in H-MCs. However, to date, there are no reports on Rpph1 in DN. Therefore, the function of Rpph1 and its molecular mechanisms in DN inflammation are unknown.

The aim of the present study was to investigate whether lncRNAs are mechanistically involved in inflammation and MC proliferation in DN. As discussed above, our data led to the identification of Rpph1 as a likely regulator of inflammation and MC proliferation by interacting with the DN-related factor galectin-3 (Gal-3). Our results provide novel insight into the mechanisms underlying inflammation and proliferation of MCs in DN, and could serve as a stepping stone for the development of intervention and prevention approaches for DN.

## Results

### LncRNA Rpph1 is significantly up-regulated in vivo and in vitro

**Fig. 1:**
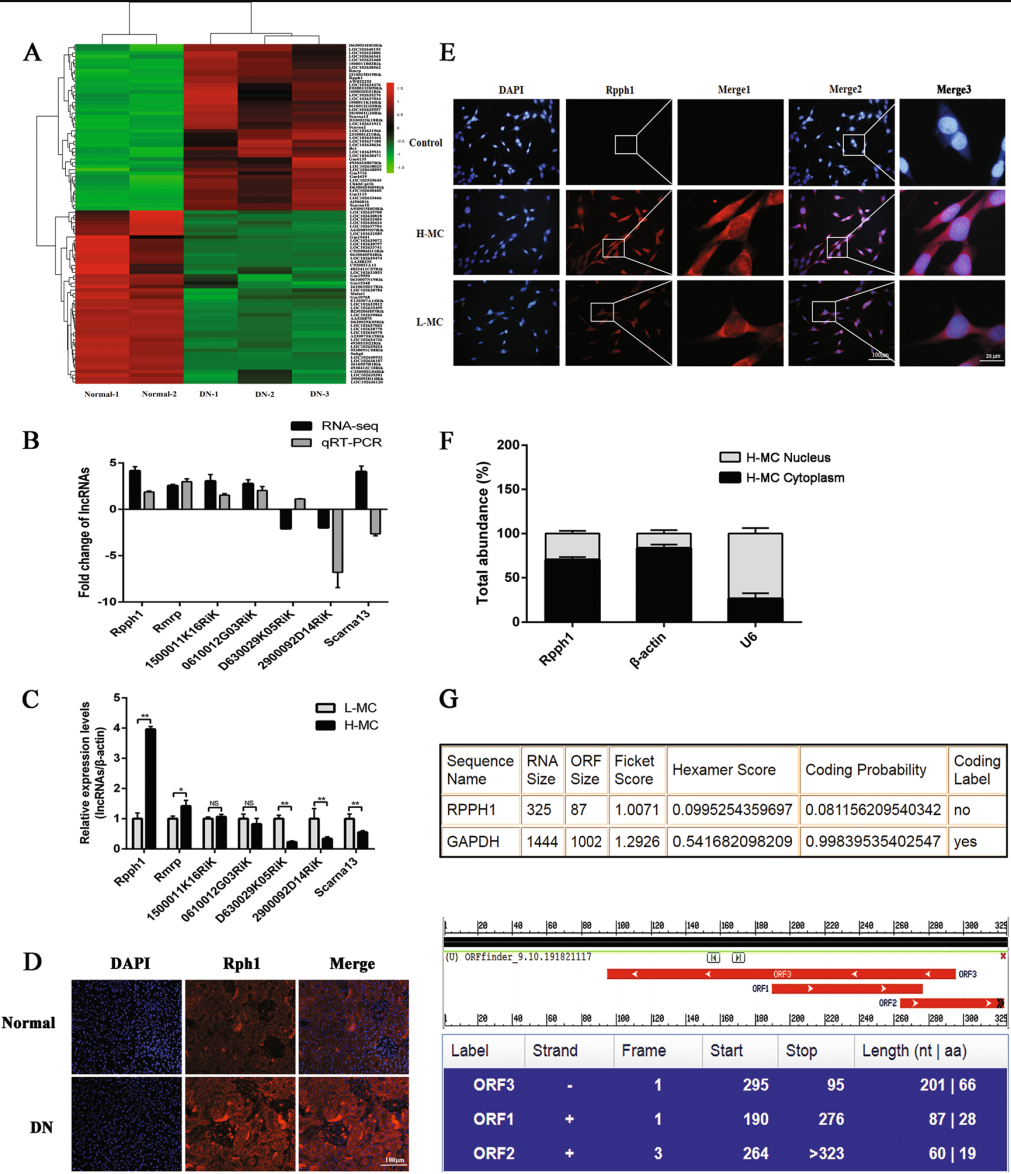
Increased expression of Rpph1 (ribonuclease P RNA component H1) was observed in diabetic nephropathy (DN) both in vivo and in vitro. **a** Dysexpressed long non-coding RNAs (lncRNAs) were examined in the renal tissue of db/db DN mice (*n* = 3 for 12 weeks) and normal controls (*n* = 2 for 12 weeks) by RNA-sequencing (RNA-seq) (*q* < 0.05). **b** Comparison of the expression of seven putative lncRNAs identified by RNA-seq and quantitative real-time PCR (qRT-PCR). The samples for qRT-PCR were the renal tissues of db/db DN mice (*n* = 3 for 12 weeks) and normal controls (*n* = 3 for 12 weeks); the data are representative of three independent experiments. **c** The seven putative lncRNA candidates were detected by qRT-PCR in mesangial cells (MCs) cultured under low (5.5 mmol/L glucose) or high glucose (25 mmol/L glucose) conditions; the data are representative of three independent experiments. Data are presented as mean ± SD. *P < 0.05, **P < 0.01, NS no significant difference. **d** Rpph1 exhibited higher expression in the renal tissue of db/db DN mice (*n* = 3 for 12 weeks) than in the normal group of mice (*n* = 3 for 12 weeks) as identified by fluorescence in situ hybridization (FISH). The data are representative of three independent experiments. **e** Rpph1 (Red) was mainly distributed in the cytoplasm of MCs cultured under low (5.5 mmol/L glucose) or high glucose (25 mmol/L glucose) conditions, as identified by FISH (×400). Merge 1: the enlarged cutouts without 4′,6-diamidino-2-phenylindole (DAPI); Merge 3: the enlarged cutouts with DAPI. **f** Rpph1 was mainly observed in the cytoplasm of MCs cultured under high glucose (25 mmol/L glucose) conditions by qRT-PCR; β-actin was the cytoplasm control and U6 was the nucleus control. The data are representative of three independent experiments. **g** The protein-coding ability of Rpph1 was predicted by bioinformatics methods. Upper: Coding-Potential Assessment Tool (CPAT) was used to test the protein-coding ability of Rpph1. Lower: ORF finder was used to analyze the protein-coding ability of Rpph1

To understand the relationship between lncRNAs and DN, we examined the lncRNAs in the renal tissue of db/db DN mice and in normal controls using RNA-seq. The data revealed that 2766 lncRNAs were detected and 95 lncRNAs were dysexpressed in DN, 46 of these were up-regulated and 49 were down-regulated (Fig. 1a and Supplementary Table 1). Among these lncRNAs, seven lncRNA candidates (FPKM (fragments per kilobase of transcript per million) >150, log 2 fold changes (log 2FC) >1), including five up-regulated and two down-regulated lncRNAs, were further verified by qRT-PCR in the renal tissue of db/db DN mice and normal controls. The data revealed that Rpph1, Rmrp, 1500011K16Rik, 0610012G03Rik, and 2900092D14Rik displayed expression consistent with the result of RNA-seq (Fig. 1b). The expressions of these candidate lncRNAs were detected in MCs under high or low glucose conditions (L-MCs) using qRT-PCR (Fig. 1c). Data showed that lncRNA Rpph1 showed the most up-regulated expression in the H-MCs and the renal cortex of db/db DN mice. Therefore, we focused on lncRNA Rpph1 for further study.

Furthermore, to explore the biological characteristics of Rpph1 in DN, its subcellular location in cells and its protein-coding ability were detected by fluorescence in situ hybridization (FISH), qRT-PCR, and bioinformatics methods. Results showed that the expression of Rpph1 was higher in the renal tissue of DN mice than in the normal group of mice (Fig. 1d). Moreover, FISH and qRT-PCR results showed that Rpph1 was expressed in both the nucleus and the cytoplasm of the L-MCs and H-MCs, but the expression of Rpph1 was markedly higher in the cytoplasm of MCs than in the nucleus (Fig. 1e, f). In addition, the non-coding nature of lncRNA Rpph1 was confirmed by coding-potential analysis (Fig. 1g). The protein-coding ability of Rpph1 was predicted by Coding-Potential Assessment Tool (CPAT) and ORF finder, and the amino acid sequences predicted by ORF finder were compared in NCBI. The results showed that the coding probability of Rpph1 was 0.08. This indicates that Rpph1 has little coding potential. In summary, all of the above results suggest that Rpph1 may have a role in the cytoplasm of MCs.

### Rpph1 promotes cell proliferation and the expression of inflammatory factors in MCs

We amplified the full length of Rpph1 and cloned its full-length sequence into a pcDNA3.1 vector to construct a stable Rpph1 over-expression plasmid, Rpph1(+). The plasmid was confirmed by gel electrophoresis and sequencing after restriction enzyme digestion (Supplementary Fig. S1A). Moreover, three small interfering RNAs (siRNAs) (siRpph1 No. 1, No. 2, and No. 3) of Rpph1 were commercially synthesized. The efficiencies of Rpph1 over-expression and knockdown were detected by qRT-PCR. The results showed that Rpph1 was significantly over-expressed in L-MCs transfected with Rpph1(+) plasmid and down-expressed in H-MCs transfected with Rpph1 siRNAs (Supplementary Fig. S1B). As shown in Supplementary Fig. S1B, qRT-PCR revealed that the knockdown effect of siRpph1 No. 2 and No. 3 were better relative to siRpph1 No.1. Thus, we used siRpph1 No. 2 and No. 3 for further experiments, and siRpph1 No. 3 was used in experiments that only use one siRNA (hereafter referred to as siRpph1). These results indicate that Rpph1 over-expression and Rpph1 knockdown were successfully performed.

**Fig. 2:**
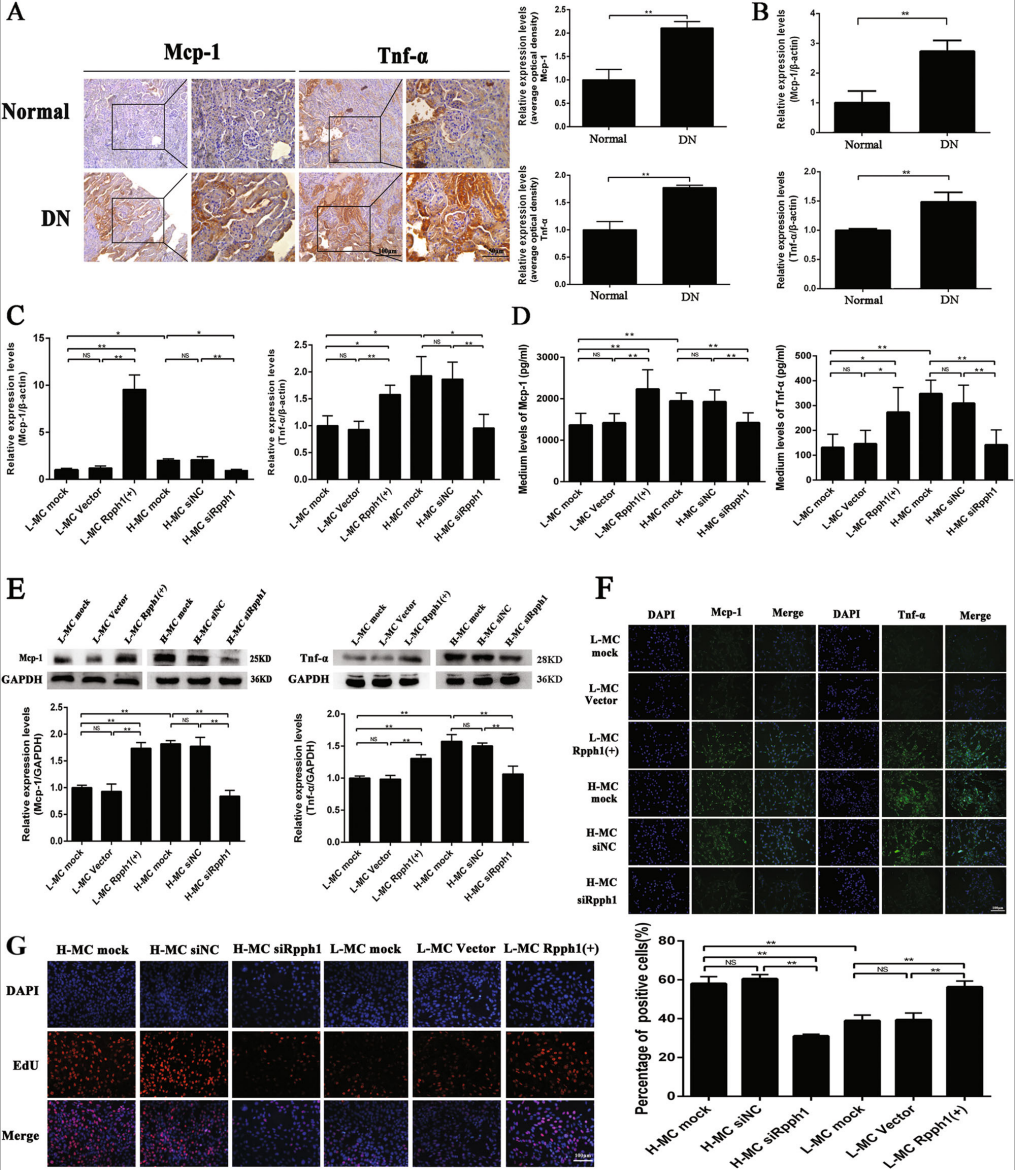
Rpph1 (ribonuclease P RNA component H1) regulated inflammation and mesangial cell (MC) proliferation. **a** The expression levels of monocyte chemoattractant protein-1 (Mcp-1) and tumor necrosis factor-α (Tnf-α) in the renal tissue of db/db diabetic nephropathy (DN) mice (*n* = 3 for 12 weeks) and normal controls (*n* = 3 for 12 weeks) were assessed by immunohistochemistry and quantitative analysis. **b** Messenger RNA (mRNA) expression levels of Mcp-1 and Tnf-α in the renal tissue of db/db DN mice and normal controls were assessed by quantitative real-time PCR (qRT-PCR). **c** Twenty-four hours after transfection of Rpph1 over-expression plasmid in L-MC, or Rpph1 small interfering RNA (siRNA) in H-MC, the expression levels of Mcp-1 mRNA and Tnf-α mRNA were detected by qRT-PCR. **d** Forty-eight hours after transfection of Rpph1 siRNA in H-MC, or over-expression plasmid in L-MC, the expression levels of Mcp-1 and Tnf-α were assessed by enzyme- linked immunosorbent assay (ELISA). **e** Forty-eight hours after transfecting Rpph1 siRNA in H-MC, or over-expression plasmid in L-MC, the expression levels of Mcp-1 and Tnf-α were assessed by Western blot and quantitative analysis. **f** Forty-eight hours after transfecting Rpph1 siRNA in H-MC, or over-expression plasmid in L-MC, the expression levels of Mcp-1 and Tnf-α were detected by immunofluorescence. **g** Forty-eight hours after transfecting Rpph1 siRNA in H-MC, or over-expression plasmid in L-MC, cell proliferation rates were detected by EdU (5-ethynyl-2′-deoxyuridine) and quantitative analysis. In all panels, the data are representative of three independent experiments. Data are presented as mean ± SD. *P < 0.05, **P < 0.01, NS not significant

Furthermore, immunohistochemistry results (Fig. 2a) and qRT-PCR (Fig. 2b) showed that expression of the pro-inflammatory cytokines Mcp-1 and Tnf-α was significantly higher in the renal tissues of DN mice than in the normal group of mice. Interestingly, the expression of Mcp-1 and Tnf-α could be regulated by Rpph1. The results of qRT-PCR (Fig. 2c), enzyme- linked immunosorbent assay (ELISA) (Fig. 2d), Western blot (Fig. 2e), and immunofluorescence (Fig. 2f) indicated that the levels of Mcp-1 and Tnf-α were significantly increased by Rpph1 over-expression in L-MCs, while the expression of Mcp-1 and Tnf-α was decreased by siRpph1 in H-MCs. Moreover, EdU (5-ethynyl-2′-deoxyuridine) assay and quantitative analysis revealed that cell proliferation in the L-MC Rpph1 (+) group was significantly increased compared with that in the L-MC vector group and the L-MC mock group. On the other hand, cell proliferation in the H-MC siRpph1 group was clearly decreased compared with the H-MC siRNA negative control (siNC) group and the H-MC mock group (Fig. 2g). Therefore, these data suggest that Rpph1 can regulate inflammation and cell proliferation in MCs.

### Rpph1 directly interacts with Gal-3

**Fig. 3:**
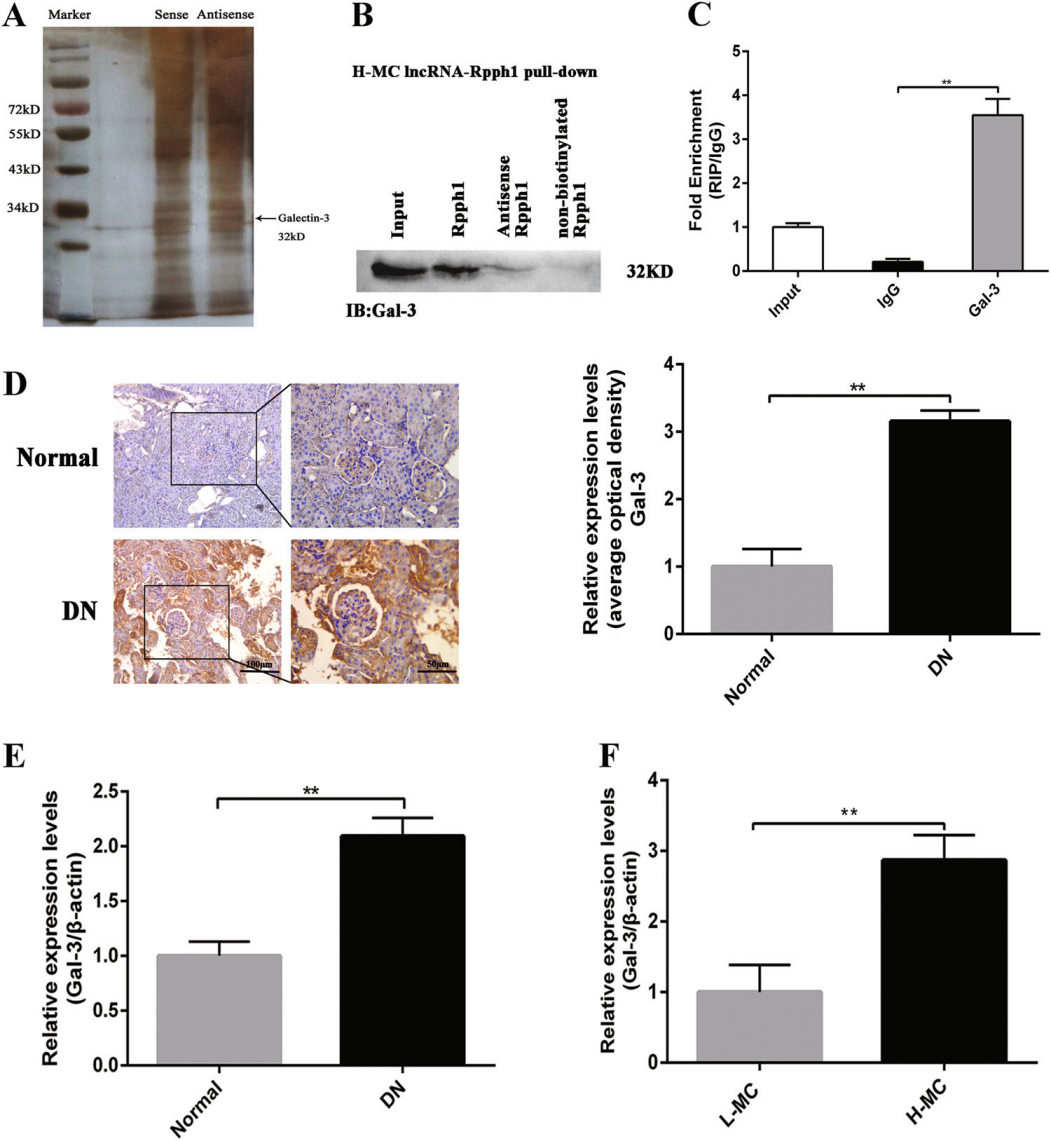
Rpph1 (ribonuclease P RNA component H1) interacted with Gal-3. **a** RNA pull-down assay was performed to detect the proteins interacting with Rpph1; mesangial cell (MC) lysates cultured under high glucose conditions were incubated with 5′ end biotin-labeled sense, antisense lncRNA Rpph1 (negative control), or non-biotin-labeled lncRNA Rpph1, and mass spectrometry and silver staining were performed to identify the interacting proteins. Black arrow: the Gal-3 band. **b** Gal-3 was detected in biotin-labeled sense, antisense lncRNA Rpph1, and non-biotin-labeled lncRNA Rpph1 groups by Western blot after pull-down; the data are representative of three independent experiments. Data are presented as mean ± SD. ^**^P < 0.01. **c** The enrichment ratio of Rpph1 with Gal-3 antibody compared with immunoglobulin G (IgG), as determined by RNA immunoprecipitation (RIP) assay; the data are representative of three independent experiments. Data are presented as mean ± SD. ^**^P < 0.01. **d** The protein levels of Gal-3 in renal tissues of diabetic nephropathy (DN) mice and normal controls was detected by immunohistochemistry and quantitative analysis; the data are representative of three independent experiments. Data are presented as mean ± SD. **P < 0.01. **e** The expression levels of Gal-3 in the renal tissue of DN mice and normal controls were detected by quantitative real-time PCR (qRT-PCR); the data are representative of three independent experiments. Data are presented as mean ± SD. **P < 0.01. **f** The expression of Gal-3 in H-MC and L-MC was detected by qRT-PCR; the data are representative of three independent experiments. Data are presented as mean ± SD. **P < 0.01

It is known that lncRNAs can perform their functions through binding to proteins in order to regulate the expression of downstream genes^28^. Therefore, we used RNA pull-down assays, mass spectrometry, and silver staining to identify lncRNA Rpph1–protein interactions in H-MCs labeled with sense lncRNA Rpph1 and antisense lncRNA Rpph1 (Fig. 3a). In total, 86 proteins were found to bind to Rpph1, including the DN-related factor Gal-3 (Supplementary Table 2). Moreover, Gal-3 was detected in the biotin-labeled sense lncRNA Rpph1 group through Western blot; the result revealed that Rpph1 directly interacts with Gal-3 (Fig. 3b). Furthermore, RNA immunoprecipitation (RIP) assay was performed using anti-Gal-3 in H-MCs to examine the relationship between Rpph1 and Gal-3. The results revealed that Rpph1 immunoprecipitated with Gal-3 antibody was enhanced relative to immunoglobulin G (IgG) control (Fig. 3c). Therefore, these data suggest that Rpph1 directly binds to Gal-3.

Since Gal-3 is a DN biomarker that is highly expressed in the serum^29,30^, we detected the expression of Gal-3 in DN in vivo and in vitro. Immunohistochemistry (Fig. 3d) and qRT-PCR (Fig. 3e) results showed that the expression of Gal-3 in the renal tissue of DN mice was obviously higher than the normal group. qRT-PCR (Fig. 3f) data also showed that the expression of Gal-3 was increased in the cells of the H-MC group. Thus, these results suggest that Rpph1 may play a role in DN via the DN-related factor Gal-3.

### Gal-3 promotes the expression of DN inflammatory factors and the proliferation of MCs via the Mek/Erk pathway

To investigate the function of Gal-3 in DN, the over-expression plasmid and siRNAs of Gal-3 were examined. We construct a stable Gal-3 over-expression plasmid Gal-3(+). The plasmid was confirmed by gel electrophoresis and sequencing after restriction enzyme digestion (Supplementary Fig. S2A). Moreover, three siRNAs (siGal-3 No.1, No. 2, and No. 3) of Gal-3 were commercially synthesized. The efficiencies of Gal-3 over-expression and knockdown were detected by qRT-PCR. The results showed that Gal-3 was significantly over-expressed in L-MCs transfected with Gal-3(+) plasmid and down-expressed in H-MCs transfected with Gal-3 siRNAs (Supplementary Fig. S2B). As shown in Supplementary Fig. S2B, qRT-PCR revealed that the knockdown effect of siGal-3 No.1 and siGal-3 No. 2 was more significant, as compared to siGal-3 No. 3. Thus, we used siGal-3 No. 1 and No. 2 for further experiments and siGal-3 No. 2 was used in experiments that only use one siRNA (hereafter termed as siGal-3). These results indicate that Gal-3 over-expression and siGal-3 knockdown were successfully performed.

**Fig. 4:**
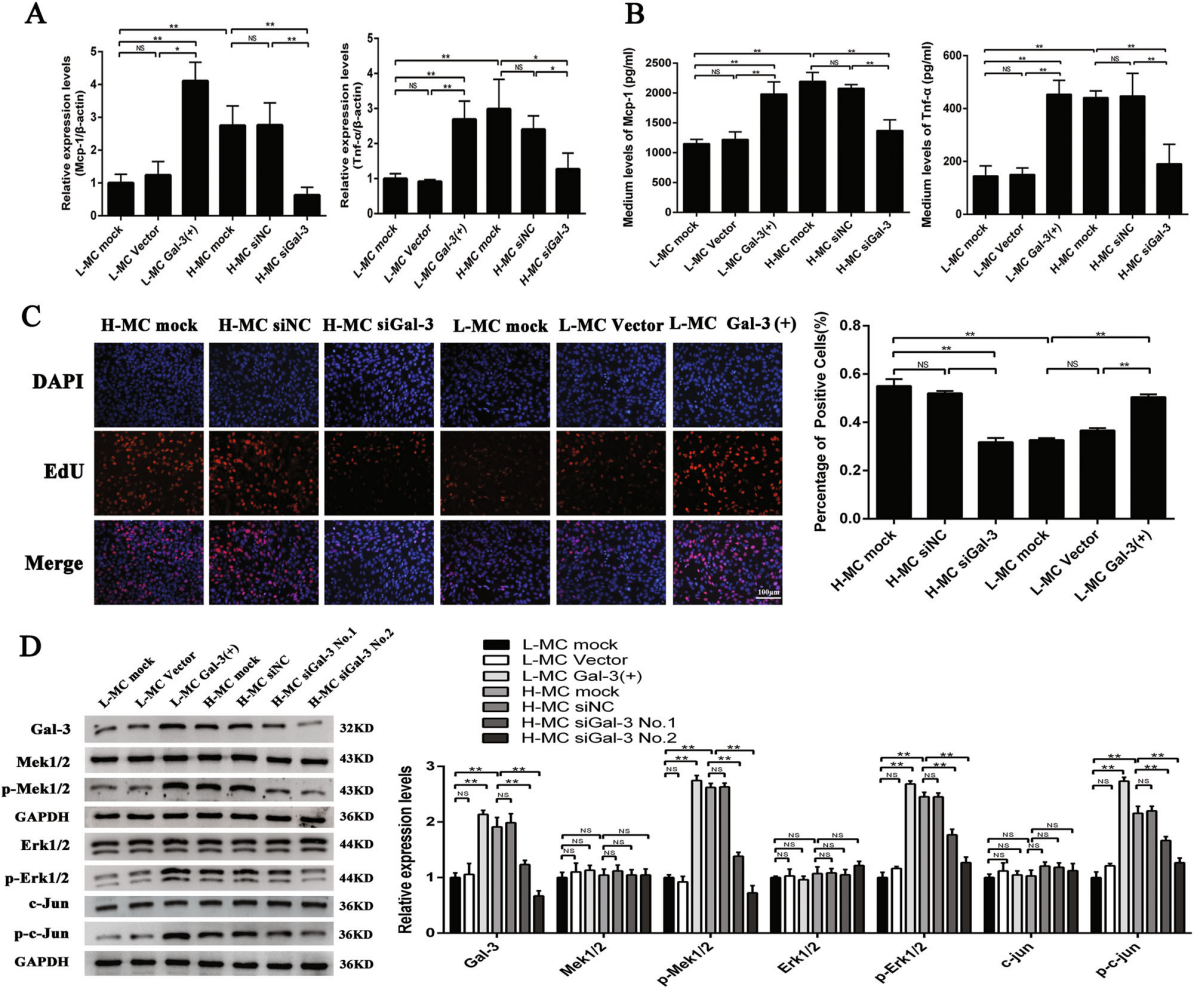
Gal-3 promotes the expression of pro-inflammatory cytokines and the proliferation of mesangial cells (MCs). **a** Twenty-four hours after transfecting Gal-3 over-expression plasmid in L-MC or Gal-3 siRNA in H-MC, the messenger RNA (mRNA) levels of monocyte chemoattractant protein-1 (Mcp-1) and tumor necrosis factor-α (Tnf-α) were detected by quantitative real-time PCR (qRT-PCR). **b** Forty-eight hours after transfecting Gal-3 over-expression plasmid in L-MC or Gal-3 siRNA in H-MC, the protein levels of Mcp-1 and Tnf-α were detected by enzyme-linked immunosorbent assay (ELISA). **c** Forty-eight hours after transfecting Gal-3 over-expression plasmid in L-MC or Gal-3 siRNA in H-MC, MC proliferation was detected by EdU (5-ethynyl-2′-deoxyuridine) assay and quantitative analysis. **d** Forty-eight hours after transfecting Gal-3 over-expression plasmid in L-MC or Gal-3 siRNA in H-MC, the expression of several key factors of the Gal-3/Mek/Erk pathway, including Gal-3, Mek1/2, p-Mek1/2, Erk1/2, p-Erk1/2, c-Jun, and p-c-Jun, were detected by Western blot and quantitative analysis. The antibodies of Mek, Erk, and c-Jun were stripped after the development by the antibody stripping solution. Then, the antibodies of p-Mek, p-Erk, and p-c-Jun were incubated with these bands for re-blotting, respectively. In all panels, the data are representative of three independent experiments. Data are presented as mean ± SD. *P < 0.05, **P < 0.01, NS not significant

Further, the expression of Mcp-1 and Tnf-α and MC proliferation was tested. qRT-PCR (Fig. 4a) and ELISA (Fig. 4b) indicated that the expression of Mcp-1 and Tnf-α was significantly increased by Gal-3 over-expression in L-MCs, while the expression of Mcp-1 and Tnf-α was decreased by siGal-3 in H-MCs. Moreover, EdU assay and quantitative analysis revealed that cell proliferation in the L-MC Gal-3(+) group was significantly increased compared with that of the L-MC vector group and the L-MC mock group. On the other hand, cell proliferation in the H-MC siGal-3 group was clearly decreased compared with the H-MC siNC group and the H-MC mock group (Fig. 4c). Therefore, these data suggest that Gal-3 may play an important role in inflammation and cell proliferation in MCs.

As recent studies have shown that Gal-3 influences the occurrence and development of DN via the Mek/Erk pathway^31^, we investigated the relationship between Gal-3 and Mek/Erk in DN in this study. The results of Western blot showed that the phosphorylation of Mek1/2, Erk1/2, and c-Jun was increased when Gal-3 was over-expressed in the L-MCs, while the phosphorylation of Mek1/2, Erk1/2, and c-Jun was decreased when Gal-3 was silenced in the H-MCs (Fig. 4d). Therefore, these results suggest that Gal-3 may participate in inflammation and proliferation of MCs in DN through the Mek/Erk pathway.

### Rpph1 regulates the expression of key factors in the Gal-3/Mek/Erk pathway in MCs

**Fig. 5:**
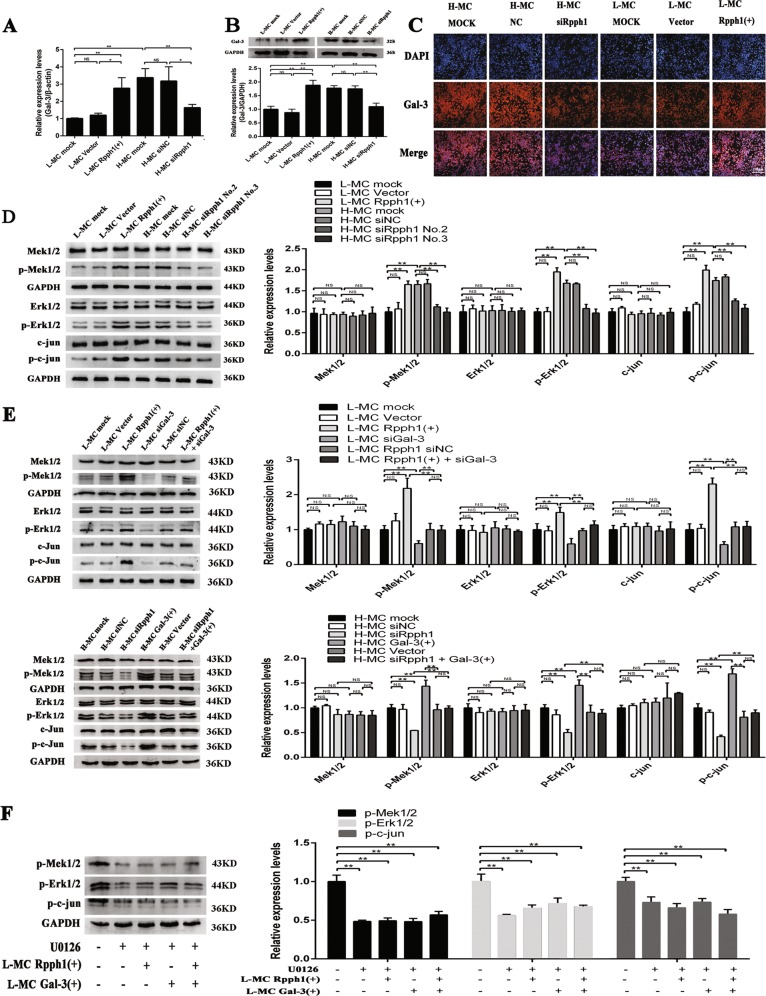
Rpph1 (ribonuclease P RNA component H1) regulates activation of the Gal-3/Mek/Erk signaling pathway in mesangial cells (MCs). **a** Twenty-four hours after transfecting Rpph1 small interfering RNA (siRNA) in H-MC or over-expression plasmid in L-MC, the messenger RNA (mRNA) levels of Gal-3 were detected by quantitative real-time PCR (qRT-PCR). **b** Forty-eight hours after transfecting Rpph1 siRNA in H-MC or over-expression plasmid in L-MC, Western blot and quantitative analysis were performed to analyze the protein levels of Gal-3. **c** Forty-eight hours after transfecting Rpph1 siRNA in H-MC or over-expression plasmid in L-MC, immunofluorescence was performed to analyze the protein level of Gal-3. **d** Forty-eight hours after transfecting Rpph1 siRNA in H-MC or over-expression plasmid in L-MC, Western blot and quantitative analysis were performed to analyze the expression of the key factors of the Mek/Erk signaling pathway. The antibodies of Mek, Erk, and c-Jun were stripped after the development by the antibody stripping solution. Then, the antibodies of p-Mek, p-Erk, and p-c-Jun were incubated with these bands for re-blotting, respectively. **e** The rescue experiment was performed to detect the effect of Rpph1 on activation or inhibition of the Mek/Erk signaling pathway. Up: Forty-eight hours after transfection with Rpph1 over-expression plasmid or co-transfection of Rpph1 over-expression plasmid and Gal-3 siRNA in L-MC, the expression of key factors of the Mek/Erk pathway were detected by Western blot and quantitative analysis. Low: Forty-eight hours after transfection with Rpph1 siRNA or co-transfection of Rpph1 siRNA and Gal-3 over-expression plasmid in H-MC, the expression of key factors of the Mek/Erk pathway were detected by Western blot and quantitative analysis. The antibodies of Mek, Erk, and c-Jun were stripped after the development by the antibody stripping solution. Then, the antibodies of p-Mek, p-Erk, and p-c-Jun were incubated with these bands for re-blotting, respectively. **f** Twenty-four hours after treatment with U0126 (10 μM) in L-MC, Western blot, and quantitative analysis were performed to analyze the effect of Rpph1 on regulation of the activation of Mek/Erk pathway; regulation was interrupted by U0126.The antibody p-Mek and glyceraldehyde-3-phosphate dehydrogenase (GAPDH) was stripped after the development by antibody stripping solution and then the antibodies of p-Erk and p-c-Jun was incubated with the bands, respectively. In all panels, the data are representative of three independent experiments. Data are presented as mean ± SD. *P < 0.05, **P < 0.01, NS not significant

Given the interaction between Rpph1 and Gal-3, we explored the effect of Rpph1 on the Gal-3/Mek/Erk pathway in L-MCs or H-MCs. qRT-PCR indicated that messenger RNA (mRNA) expression of Gal-3 was regulated by Rpph1 (Fig. 5a). Moreover, Western blot (Fig. 5b) and immunofluorescence (Fig. 5c) revealed that the protein expression of Gal-3 was regulated by Rpph1. In addition, to investigate the effect of Rpph1 on the Mek/Erk signaling pathway, we used Western blot to detect the expression of key factors in the Mek/Erk pathway. The results demonstrated that over-expression of Rpph1 could promote the phosphorylation of Mek1/2, Erk1/2, and c-Jun, while Rpph1 knockdown significantly compromised the phosphorylation of Mek1/2, Erk1/2, and c-Jun (Fig. 5d). Further, the results of Western blot revealed that the effect of over-expression of Rpph1 on the activation of Mek/Erk pathway could be rescued by silencing of Gal-3, while the effect of Rpph1 knockdown on inhibition of Mek/Erk pathway could be rescued by over-expression of Gal-3 (Fig. 5e). Additionally, U0126 inhibited the activation of the Mek/Erk pathway. As known, U0126 is a widely used inhibitor that inhibits multiple key kinases of MAPK signaling pathway, including Mek1/2, Mek5, Erk5, and so on^32^. The effect of over-expression of Rpph1 or Gal-3 on the activation of the Mek/Erk pathway was interrupted by U0126 (Fig. 5f). These results suggest that Rpph1 may play a role in the Gal-3/Mek/Erk pathway in DN.

### Rpph1 regulates inflammation and proliferation of MCs by activating the Gal-3/Mek/Erk pathway in DN

**Fig. 6:**
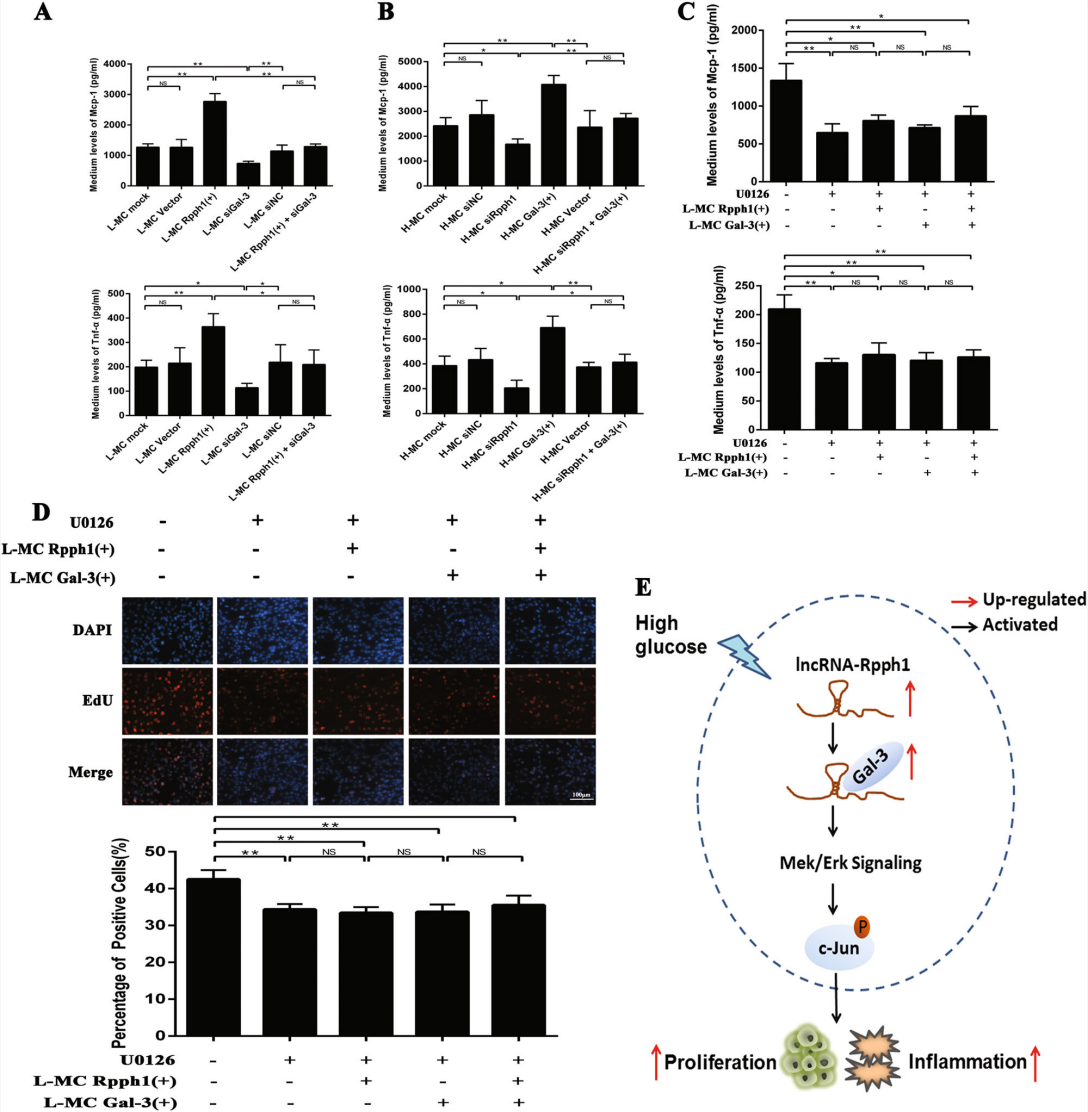
Rpph1 (ribonuclease P RNA component H1) promotes the expression of pro-inflammatory cytokines and cell proliferation via the Gal-3/Mek/Erk pathway. **a** Forty-eight hours after transfection with Rpph1 over-expression plasmid or Rpph1 over-expression plasmid and Gal-3 small interfering RNA (siRNA) in L-MC, enzyme- linked immunosorbent assay (ELISA) was performed to analyze the effect of Rpph1 on the expression of tumor necrosis factor-α (Tnf-α) and monocyte chemoattractant protein-1 (Mcp-1) with Gal-3 knockdown. **b** Forty-eight hours after transfection with Rpph1 siRNA or Rpph1 siRNA and Gal-3 over-expression plasmid in H-MC, ELISA was performed to analyze the effect of Rpph1 on the expression of Tnf-α and Mcp-1 with Gal-3 over-expression. **c** Twenty-four hours after treatment with U0126 (10 μM) in L-MC, ELISA was performed to analyze the effect of over-expression of Rpph1 or Gal-3 on the expression of Mcp-1 and Tnf-α with U0126; U0126 inhibited the expression of inflammatory cytokines relative to the untreated group. In addition, the effect of over-expression of Rpph1 or Gal-3 on promotion of the expression of inflammatory cytokines was blocked by U0126 in L-MC. **d** Twenty-four hours after treatment with the U0126 (10 μM) in L-MC, EdU (5-ethynyl-2′-deoxyuridine) assay was performed to analyze the blocking effect of over-expression of Rpph1 or Gal-3 on cell proliferation with U0126; U0126 inhibited cell proliferation relative to the untreated group and blocked the effect of over-expression of Rpph1 or Gal-3 on cell proliferation. **e** The abridged general view of the mechanism whereby lncRNA Rpph1 promotes the expression of Mcp-1 and Tnf-α and the proliferation of mesangial cells (MCs) in DN via an interaction with Gal-3 and activation of Gal-3/Mek/Erk signaling. In all panels, the data are representative of three independent experiments. Data are presented as mean ± SD. *P < 0.05, **P < 0.01, NS not significant

In view of the above results on the relationship between Rpph1 and the Gal-3/Mek/Erk pathway in MCs, a further rescue experiment was performed to investigate the mechanism by which Rpph1 influences inflammation and proliferation of MCs via the Gal-3/Mek/Erk pathway. The results of the ELISA analysis revealed that the effect of Rpph1 over-expression on increasing the expression of Mcp-1 and Tnf-α was inhibited by Gal-3 knockdown (Fig. 6a). Moreover, the effect of siRpph1 on decreasing the expression of Mcp-1 and Tnf-α was rescued by the over-expression of Gal-3 (Fig. 6b). U0126 inhibited cell proliferation and the expression of inflammatory cytokines relative to the untreated group; the ELISA analysis also showed that the effect of over-expression of Rpph1 or Gal-3 on increasing the expression of Mcp-1 and Tnf-α was blocked by U0126 (Fig. 6c). In addition, EdU revealed that the effect of over-expression of Rpph1 or Gal-3 on the promotion of cell proliferation was blocked by U0126 in MCs (Fig. 6d).

In addition, siRNAs of Mek1 (siMek1 No. 1, No. 2, and No. 3) and Mek2 (siMek1 No. 1, No. 2, and No. 3) were commercially synthesized, and the knockdown efficiency of the siRNAs was detected by qRT-PCR (Supplementary Fig. S3A). The knockdown effect of siMek1 No. 1 and No. 3 and siMek2 No. 1 and No. 2 was better relative to the other siRNA. Thus, we used siMek1 No. 1 and No. 3 and siMek2 No. 1 and No. 2 for further experiments and the siMek1 No. 1 and siMek2 No. 2 were used for experiments that only use one siRNA (hereafter termed as siMek1 and siMek2, respectively). The results of Western blot indicated that the expression of Mek1 was obviously knocked by siMek1 and the expression of Mek2 was significantly knocked by siMek2 (Supplementary Fig. S3B). Both Mek1 and Mek2 knockdown significantly compromised the phosphorylation of Erk1/2 and c-jun (Supplementary Fig. S3B). The results of ELISA showed that knockdown of both Mek1 and Mek2 decreased the expression of Mcp-1 and Tnf-α (Supplementary Fig. S3C). It can be seen from Supplementary Fig. S3D that the proliferation of MCs was suppressed after transfection of siMek1 and siMek2. Taken together, these results suggest that Rpph1 may regulate inflammation and the proliferation of MCs in DN by activating the Gal-3/Mek/Erk pathway (Fig. 6e).

## Discussion

In this study, we explored the role of the lncRNA Rpph1 in MCs in facilitating the progression of DN. Rpph1 was found to be significantly increased in both the renal tissue of DN mice and in H-MCs. Moreover, Rpph1 promoted the expression of Mcp-1 and Tnf-α and the proliferation of MCs by directly interacting with DN-related factor Gal-3 and activating the downstream pathway of the Mek/Erk signaling pathway. In brief, the results of this study reveal a novel mechanism underlying the progression of inflammation in DN.

LncRNAs are extensively involved in biological processes such as histone modification, DNA methylation, and chromatin remodeling^33^. Wang et al.^34^ reported that over-expression of lncRNA-CYP4B1-PS1-001 inhibited the proliferation and fibrosis of MCs. Further, lncRNAs also participate in DN inflammation. Carpenter et al.^35^ reported that lincRNA-cox2 plays a role in promoting and inhibiting the expression of inflammatory factors. Several lncRNAs, including THRIL^36^ and lnc13^37^, have been reported to regulate inflammatory gene expression in myeloid cells. Thus, the literature suggests that lncRNAs may participate in the development of inflammation in DN. However, to date, the exact mechanism remains unclear. An in-depth understanding of the role of target lncRNAs in DN inflammation is crucial for understanding the mechanism underlying DN.

In addition, Rpph1 was found to be significantly increased in vivo and in vitro using RNA-seq and qRT-PCR. Rpph1 is known as the RNA constituent of RNase P, located on mouse chromosome 14 (Chr12:21,417,911–21,419,803) and having a length of 325 bp. Recent studies have shown that Rpph1 is up-regulated in both the human gastric cancer tissue^38^ and the neocortex of patients with epilepsy^39^. Zhang and Tang^40^ reported that Rpph1 knockdown inhibits the proliferation of breast cancer cells and the occurrence of tumors^40^, and Rpph1 was found to promote hippocampal neuron dendritic spine formation^41^. These findings suggest that Rpph1 may be an important factor in disease. Our results show that Rpph1 regulates MC proliferation and the expression of pro-inflammatory cytokines Tnf-α and Mcp-1. Therefore, our findings suggest that increased expression of Rpph1 may represent a potential risk for renal inflammation and MC proliferation, and may contribute to the development and progression of DN.

LncRNAs have various functions depending on their subcellular location. Our data showed that Rpph1 is mainly a cytoplasmic lncRNA in MCs, indicating that it exerts its biological effects primarily by interacting with RNA or binding to proteins^42,43^. It is known that the interactions between lncRNAs and proteins play a role in various diseases, including DN. One report indicated that lncRNA-Tug1 interacts with PGC-1α at its promoter region, resulting in elevated Ppargc1a transcriptional output and regulation of mitochondrial bioenergetics in DN^44^. Further, lncRNA PVT1 promotes LPS-induced septic acute kidney injury by binding to TNF-α^45^. These studies suggest that the mechanism of lncRNA–protein complex formation plays an important role in DN. Therefore, we focused on the mechanism of Rpph1 and its related proteins in the current study. Our data showed that the DN-related factor Gal-3 directly binds to Rpph1. Gal-3, a member of the lectin family, has a conserved amino acid sequence and can specifically identify the ion-galectin structure^46^. Gal-3 may be an indicator for the early detection of prediabetes and diabetes^47^. Serum Gal-3 is independently associated with progression of nephropathy in type 2 diabetes mellitus^48^. Moreover, our results revealed increased expression of Gal-3 in the renal tissues of DN mice and in H-MCs. These results suggest that Gal-3 may participate in DN. In addition, studies have shown that Gal-3 is involved in the inflammatory response^49^. Gal-3 deficiency results in significantly reduced peritoneal inflammatory responses to thioglycollate stimulation^50^. Further, Gal-3 was found to be a potentiator of skin inflammation in a mouse model of AD^51^. Several studies have also indicated that a hyperglycemic environment could induce excessive expression of Gal-3; the over-expression of Gal-3 induces activation of mast cells, thereby regulating inflammation^52,53^. Furthermore, our results showed that Gal-3 increased the expression of pro-inflammatory cytokines and increased proliferation of MCs. Therefore, these results suggest that Rpph1 regulates inflammation and proliferation of MCs by directly interacting with Gal-3.

Song et al.^54^ reported that Gal-3 activates the Mek/Erk signaling pathway^54^. The Mek/Erk signaling pathway is reported to be involved in the Gal-3-mediated invasiveness of osteosarcoma cells^55^. Further, Gal-3 modulates uPAR expression via the Mek/Erk pathway to inhibit the proliferation and invasion of hepatocellular carcinoma cells^56^. Therefore, the Mek/Erk signaling pathway was a focus in the current study. Our data showed that Rpph1 increases the expression of Gal-3, and activated the Mek/Erk signaling pathway. Moreover, the effect of Rpph1 on the activation of the Mek/Erk pathway could be rescued by the knockdown of Gal-3. Additionally, the effect of Rpph1 or Gal-3 on inflammation and MC proliferation could be blocked by U0126. In order to further investigate the role of Mek in MCs, we transfected the Mek siRNAs in H-MCs, and the results of siRNAs and inhibitor experiments showed the similar effects. Taken together, these results indicate that the Gal-3/Mek/Erk signaling pathway could be the potential mechanism underlying the effect of Rpph1 on inflammation and proliferation of MCs in DN.

In conclusion, the present study identified that Rpph1 may play a critical role in inflammation and proliferation of MCs in DN. Our results further revealed that Rpph1 can directly interact with Gal-3. Moreover, we demonstrated that Rpph1 increases Gal-3 expression and activity in DN. In addition, we found that the effect of Rpph1 on the regulation of expression of pro-inflammatory cytokines and proliferation of MCs is likely to occur via the Gal-3/Mek/Erk signaling pathway. The modulation of Rpph1 may provide an intriguing approach for tackling inflammation and proliferation of renal MCs in DN.

## Materials and methods

### Animals

Naturally developed DN in mice with genetic defects in the leptin receptor (db/db) is a well-established model for type 2 diabetes, obesity, and insulin resistance. Eight-week-old male db/db mice with C57BL/BKS background (DN group) and age-matched male genetic control db/m mice (normal group) were purchased from NBRI (Nanjing, China) and raised on the Specific Pathogen-Free scale^57^. When the mice in the DN group were 12 weeks old and their body weight, urine microalbumin, and blood glucose indices were significantly higher than those in the control group (Supplementary Fig. S4), the renal tissues of the mice were extracted for further study. All procedures were performed in accordance with the institutional guidelines for the care and use of laboratory animals at Chongqing Medical University. Ethics approval was obtained from the Ethics Committee of Chongqing Medical University.

### Cell culture

The mouse MC line (SV40-MES14) was preserved in our laboratory. MCs were cultured in Dulbecco’s modified Eagle’s medium with 20% fetal bovine serum in a humidified atmosphere containing 5% CO_2_ at 37 °C. Many previous studies have indicated that high glucose conditions simulate the growth environment of mouse glomerular MCs under the state of DN, while low glucose conditions simulate a normal growth status^58^. Accordingly, cells were stimulated with d-glucose at 5.5 mmol/L glucose plus 19.5 mmol/L mannitol (low glucose group; L-MC group) or at 25 mmol/L glucose (high glucose group; H-MC group).

### Plasmids, siRNAs, U0126, and transfection

The genomic DNA of MCs was extracted and the full-length of Rpph1 was amplified; the PCR products were cloned into the pcDNA3.1 expression vector (Invitrogen, CA, USA). The Gal-3 over-expression constructs were built by cloning Gal-3 complementary DNA (cDNA) into a EX-Mm03620-M98 vector (Genecopoeia, Maryland, USA). Plasmids were extracted by an Endotoxin Removal Plasmid Extraction Kit (Omega, GA, USA) following the manufacturer’s protocol and were identified by enzyme digestion identification (Rpph1 over-expression plasmid digested by *Bam*HI and *Eco*RV; Gal-3 over-expression plasmid digested by *Bsr*GI and *Sa1*I), PCR, and sequencing (Sangon Biotech, Shanghai, China). All the plasmids in the experiment were transfected with Lipofectamine 3000 reagent (Invitrogen, CA, USA) following the manufacturer’s protocol.

Rpph1, Mek1 siRNAs, Mek2 siRNAs, and scrambled siRNA were designed and purchased from Ribo Biotechnology Co. Ltd (Guangzhou, China). Gal-3 siRNA was designed and purchased from Genecopoeia (Maryland, USA). The siRNA sequence of Gal-3 was 5′-UCAUUGUGUGUAACACGAATT-3′ (sense), 5′-UUCGUGUUACACACAAUGATT-3′ (antisense). The siRNA sequence targeting Rpph1 was 5′-CCAGCAGTGCGAGTTCAAT-3′, Mek1 was 5′-GGTTATGGCTAGAAAGCTG-3′, and Mek2 was 5′-GCTCAGACTTCCAGGAGTT-3′. All siRNAs were transfected at a 50 nM final concentration using Lipofectamine 2000 reagent (Invitrogen, CA, USA) or LipoFiter^TM^ liposomal transfection reagent (Hanbio Biotechnology, Shanghai, China), according to the manufacturer’s instructions.

The MAPK inhibitor U0126 (Selleck Chemical, TX, USA) was used for inhibiting the activation of Mek1/2 in MCs. The U0126 powder was dissolved in dDimethyl sulfoxide and the final concentration in MCs were 10 μM^59^. Cells were treated with U0126 for 24 h^60^.

The transfection was conducted using Opti-MEM, Lipofectamine 2000, and Lipofectamine 3000 reagents according to the manufacturers’ protocols. MCs in the H-MC group were transfected with Rpph1 siRNA, Gal-3 siRNA, Mek1, and Mek2 siRNA, and were named the H-MC siRpph1 group, H-MC siGal-3 group, H-MC siMek1 group, and H-MC siMek2 group, respectively. Cells in the H-MC group transfected with the siRNANC of Rpph1, Gal-3, Mek1, and Mek2 were named the H-MC siNC group, and cells in the H-MC group without transfection were named the H-MC mock group. Further, MCs in the L-MC group transfected with the over-expression plasmid of Rpph1 or Gal-3 were named the L-MC Rpph1(+) group or the L-MC Gal-3(+) group; cells in the L-MC group transfected with the empty plasmid were named the L-MC vector group, and the cells in the L-MC group without transfection were named the L-MC mock group. The transfection efficacy was measured by qRT-PCR.

### RNA-sequencing

Total RNA was extracted from the renal tissues of three mice in the DN group and three mice in the normal group with TRIzol^TM^ reagent (Invitrogen, California, USA). The purity and concentration of RNA was measured using an Agilent 2100 Bioanalyzer system (Agilent, California, USA). One sample of the normal group was rejected because of its poor quality. The three mice in the normal group showed no significant difference in gender, weight, and age. The Dynabeads® mRNA Purification Kit (Invitrogen, California, USA) was used to purify the mRNA from total RNA. The fragmentation buffer was added to break the mRNA into small pieces and the fragmented mRNA was used as the template to construct the cDNA library. The library was sequenced by Illumina HiSeq^TM^ 2000 sequencing platform of Beijing Genomics Institute (Shenzhen, China) and with read lengths of 90 bases by paired-end sequencing. Adapter sequences, low-quality (containing more than 50% of base pairs where [QA] <15) data, and data containing more than 2% unrecognized nucleotide sequences were first removed from the raw data. Processed data were annotated in UCSC mm10 genome using Tophat (v2.0.13). Cufflinks (v2.2.1) was used for combinatorial evaluation of transcriptional data and detection of differential expression with the following criteria: log 2FC >0.585 or <−0.585, and false discovery rate <0.05. The hot map based on the differentially expressed genes analysis was drawn using R. It should be noted that there was no significant difference in the RNA-seq results between the two mice in the normal group. Therefore, we only showed the data of the two normal samples and three DN samples.

### LncRNA coding-potential analysis

Coding potential was analyzed by the online software ORF finder (www.ncbi.nlm.nih.gov/orffinder/) and CPAT (Coding-Potential Assessment Tool) (http://lilab.research.bcm.edu/cpat/).

### RNA extraction and qRT-PCR analysis

Total RNA of tissues and cells was extracted by Trizol (Invitrogen, CA, USA) according to the manufacturer’s instructions. In total, 500 ng of total, cytoplasmic, and nuclear RNA was reverse-transcribed into cDNA using the PrimeScript RT Reagent Kit (Takara, Shiga, Japan). SYBR Premix Ex Taq^TM^ (Takara, Shiga, Japan) was used for qRT-PCR to detect the fold changes of lncRNAs or mRNAs. A detailed list of primer sequences is provided in Supplementary Table 3. The reaction program was as follows: 95 °C for 3 min, 95 °C for 5 s, 58 °C for 34 s, and 2 °C for 60 s. All experiments were conducted at least three times. The relative quantitative data of lncRNAs and mRNAs were analyzed by the 2^–∆∆Ct^ method and were normalized to β-actin.

Cytoplasmic and nuclear RNA was extracted from MCs using a PAKIS Kit (Thermo Fisher Scientific, Waltham, MA, USA) according to the manufacturer’s protocol. The purity and concentration of RNA was measured using a Nanodrop ND-2000 (Thermo Fisher Scientific) according to the manufacturer’s operating instructions. The nuclear control was U6 and the cytoplasmic control was β-actin^14,61^. A detailed list of primer sequences is provided in Supplementary Table 3.

### Fluorescence in situ hybridization

The lncRNA Rpph1 FISH probe was designed and synthesized by Ribo Biotechnology Co. Ltd (Guangzhou, China). The localization of Rpph1 was detected with a FISH Kit according to the manufacturer’s instructions. The cells were divided into three groups: H-MC, L-MC, and control (negative control; cells in the H-MC group treated with 4′,6-diamidino-2-phenylindole (DAPI) only). The cells were inoculated in a 24-well plate with glass slides. Briefly, after 24 h cultivation, the cells were washed four times with phosphate-buffered saline (PBS), fixed with 4% paraformaldehyde for 10 min at room temperature, and permeabilized with 0.5% Triton X-100 for 5 min. Then, cells were treated with pre-hybridization buffer at 37 °C for 30 min and were hybridized with a mixed liquor consisting of lncRNA Rpph1 probe and hybridization buffer diluted at a ratio of 1:50; hybridization was performed at 37 °C overnight and then cells were washed three times in the order of 4× saline sodium citrate (SSC), 2× SSC, and 1× SSC at 45 °C. Further, cells were treated with DAPI at room temperature for 8 min. Finally, the images were observed with a confocal microscope and were analyzed with LAS AF Lite (Leica, Solms, Germany).

### RNA pull-down and RNA immunoprecipitation assays

For RNA pull-down, in brief, the PCR primers containing the T7 promoter were synthesized and the DNA template was obtained by PCR amplification. In vitro transcription was performed using a Ribo^TM^ RNAmax-T7 biotin-labeled transcription Kit (Ribo Biotechnology Co. Ltd, Guangzhou, China). According to the specifications of the Pierce^TM^ magnetic RNA-Protein Pull-down Kit (Thermo Fisher Scientific, MA, USA), biotin-labeled RNA was co-incubated with cell lysates on the shaker overnight. The mixed RNA-protein binding reaction solution was incubated with magnetic beads for 1 h; after that, elution of RNA-binding protein complexes was executed by elution buffer at 50 °C for 40 min. Then, the eluate was collected and the proteins bound with Rpph1 were detected by mass spectrometry and silver stain. The Rpph1-bound protein Gal-3 was then tested by Western blot.

For mass spectrometry, briefly, the protein polypeptide samples were lysed by the protease, trypsin (Promega, WI, USA). Samples of enzymatic hydrolysis were analyzed by nanoLC-QE LCMSMS (Thermo Fisher Scientific, MA, USA).Then the data of LCMSMS was evaluated by Mascot Daemon v2.5.1. The identification information of target protein polypeptide was obtained from the data. The silver stain was performed by Fast Silver Stain Kit (Beyotime Bitechnology, Shanghai, China).

To determine whether Rpph1 was associated with Gal-3, RIP assays were performed using an EZMagna RIP Kit (Millipore, Billerica, MA, USA) and a Gal-3 antibody (Santa Cruz Biotechnology, USA), following the manufacturer’s protocol. qRT-PCR analysis was performed to measure the expression levels of Rpph1 and Gal-3. Normal mouse IgG (Millipore, Billerica, MA, USA) was used as a negative control.

### Western blot analysis

Cells were lysed by RIPA lysis buffer (Beyotime Bitechnology, Shanghai, China) mixed with phenylmethanesulfonyl fluoride at 4 °C for 40 min. The cell lysate was centrifuged at 15,500 × *g* for 30 min. The protein concentration in the supernatant was measured by a BCA Protein Assay Kit (Thermo Fisher Scientific, USA). The same amount of proteins was separated by sodium dodecyl sulfate-polyacrylamide gel electrophoresis, and the gel was then cut and the proteins were transferred. The membrane was then blocked with milk or bovine serum albumin (anti-phosphorylated protein) for 3 h and incubated with the primary antibody overnight at 4 °C. After that, it was incubated with the secondary antibody for 1.5 h at room temperature and an ECL Western Blotting Substrate Kit (Millipore, USA) was used to analyze the relative levels of the proteins. Glyceraldehyde-3-phosphate dehydrogenase was used as the internal control. The specific antibody dilution ratios were as follows: anti-Mcp-1 (1:2000, Abcam, Eugene, USA), anti-TNF-α (1:1000, BBI Life, Shanghai, China), anti-Gal-3 (1:500, Santa Cruz Biotechnology, USA), anti-Mek1/2 (1:3000, Abcam, Eugene, USA), anti-p-Mek1/2 (1:3000, Abcam, Eugene, USA), anti-Erk1/2 (1:1500, BBI Life, Shanghai, China), anti-p-Erk1/2 (1:1500, BBI Life, Shanghai, China), anti-c-Jun (1:1000, KleanAB, Shanghai, China), and anti-p-c-Jun (1:1000, KleanAB, Shanghai, China). The antibodies of Mek, Erk, and c-Jun were stripped after the development by the antibody stripping solution (Beyotime Bitechnology, Shanghai, China). Then, the antibodies of p-Mek, p-Erk, and p-c-Jun were incubated with these bands for re-blotting, respectively. The Image J software was used to analyze the gray value of the protein bands.

### Immunofluorescence analysis

Cells were tiled in a 24-well plate containing sterile cover glass slides and were cultured for 48 h, fixed with paraformaldehyde for 30 min, incubated with 0.3% Triton X-100 for 10 min, blocked with 3% goat serum for 1 h, and then incubated overnight with anti-Mcp-1 (1:50, Abcam, Eugene, USA), anti-Tnf-α (1:50, BBI Life, Shanghai, China), and anti-Gal-3 (1:50, Santa Cruz Biotechnology, USA). Then, cells were washed three times with PBS; DyLight488 or DyLight549 (Thermo Fisher Scientific, MA, USA) was then added into the cells for 1 h under dark conditions. Next, cells were washed with PBS three times and treated with DAPI for 10 min. Finally, the images were observed with a confocal microscope and were analyzed with LAS AF Lite (Leica, Solms, Germany).

### Immunohistochemistry

The experiment was performed in accordance with the manufacturer’s instructions; the SPlink Detection Kit was purchased from Zsgb-Bio Co. Ltd (Beijing, China). Renal tissues were fixed with 4% paraformaldehyde, embedded in paraffin, and sectioned at a thickness of 5 µm. Antigen retrieval was carried out by boiling the samples in citrate buffer for 15 min at 92–98 °C; then, the sections were treated with 3% hydrogen peroxide for 30 min to inhibit peroxidase activity and were rinsed with PBS three times. Next, 10% normal goat serum was added for 1 h at 37 °C, and subsequently, incubation was carried out with anti-Mcp-1 (1:50, Abcam, Eugene, USA), anti-Tnf-α (1:50, BBI Life, Shanghai, China), and anti-Gal-3 (1:50, Santa Cruz Biotechnology, USA) overnight at 4 °C. The sections were then incubated with biotin-labeled secondary antibody at room temperature for 30 min. The nuclei were counterstained with hematoxylin. The immunohistological staining for Mcp-1, Tnf-α, and Gal-3 was observed by light microscopy.

### Enzyme-linked immunosorbent assay

A mouse Mcp-1 Enzyme-Linked Immunosorbent Assay (ELISA) Kit and mouse Tnf-α ELISA Kit were purchased from BOSTER Biological Technology Co. Ltd (Wuhan, China); the tests were performed according to the manufacturer’s instructions for the analysis of levels of proteins in MCs. A total of 6 × 10^5^ MCs were cultivated into 6-well plates for 48 h and the medium supernatant was collected. The diluted samples and standard products of different concentrations (1000, 500, 250, 125, 62.5, 31.3, or 15.6 pg/ml) were added into the 96-well ELISA plate. Each group was provided with three parallel wells. The samples were sealed with sealing film for 90 min at 37 °C and then the Mcp-1 or Tnf-α antibody working solution was added into the plate and incubated for 60 min at 37 °C. The samples were rinsed three times with wash buffer, and then 100 μl of prepared ABC working solution was added into the plate for 30 min at 37 °C. Next, the samples were washed five times with wash buffer and stained with 3, 3′, 5, 5′-tetramethylbenzidine (TMB) substrate until there was a clear blue grading. The reaction was terminated with TMB stop buffer. Finally, the optical density value was measured at 450 nm by an enzyme microplate reader and the concentration of proteins was measured according to the standard curve.

### Cell proliferation assay

The replication of DNA was detected by an EdU incorporation assay according to the manufacturer’s protocol (Ribo Biotechnology Co. Ltd, Guangzhou, China). A total of 1.5 × 10^5^ cells were inoculated in a 24-well plate. Labeling medium was added into each well for 2 h at 37 °C under 5% CO_2_. Samples were fixed with 4% paraformaldehyde for 30 min, permeated with 0.3% Triton X-100 for 10 min, and washed with PBS. Then, the cell samples were incubated with Apollo buffer under the caliginous condition for 30 min at room temperature. Subsequently, cells were mixed with Hoechst 33,342 buffer and incubated for 30 min, and were then washed three times with PBS. Finally, images were obtained with a microscope (Olympus, Tokyo, Japan) and were analyzed with Image-Pro Plus (Media Cybernetics, Bethesda, MD, USA). The EdU incorporation rate was calculated as the ratio of EdU-positive cells (red cells) to total Hoechst 33342-positive cells (blue cells).

### Statistical analysis

Student’s *t* tests and one-way analysis of variances (no less than three groups) with Tukey’s multiple comparisons tests were performed to analyze the significance of differences between groups. *P* < 0.05 was regarded as a statistically significant difference. GraphPad Prism 6 statistical package (GraphPad Software, San Diego, USA) was used to conduct data analysis.

## Supplementary information

Supplementary data.

Supplementary Table 1.

Supplementary Table 2.

Supplementary Table 3.
