# Supplementary material for: Long non-coding RNA Rpph1 promotes inflammation and proliferation of mesangial cells in diabetic nephropathy via an interaction with Gal-3
Source: Cell Death Dis. 2019 Jul 8;10(7):526. doi: 10.1038/s41419-019-1765-0 (PMC6614467; doi:10.1038/s41419-019-1765-0)

Fig. S1. Validation of over-expression plasmid and siRNAs of Rpph1. (A) Rpph1 over-expression plasmid was verified by restriction enzyme digestion and nucleotide sequencing. Lane M: marker, Lane 1: pcDNA3.1, Lane 2: plasmid digested by *Bam*HI and *EcoR*I. (B)Twenty-four hours after transfection of Rpph1 siRNAs (50 nM) in H-MC, or over-expression plasmid (2500 ng) in L-MC, in 6-well-plates, Rpph1 over-expression and knockdown efficiency were detected by qRT-PCR. The data are representative of three independent experiments. Data are presented as mean±SD. **P*<0.05, ***P*<0.01, NS not significant.

Fig. S2. Validation of over-expression plasmid and siRNAs of Gal-3. (A) Gal-3 over-expression plasmid was verified by restriction enzyme digestion and nucleotide sequencing. Lane 1: DNA ladder 6000, Lane 2: EX-Mm03620-M98 plasmid, Lane 3: plasmid digested by BsrGI (there are three expected bands (~738/5559/858bp)), Lane 4: plasmid digested by Sa1I (there are three expected bands (~2357/2188/2610bp)). (B) Twenty-four hours after transfection of Gal-3 siRNAs (50 nM) in H-MC, or over-expression plasmid (2500 ng) in L-MC, in 6-well-plates, the Gal-3 over-expression and knockdown efficiency were detected by qRT-PCR. The data are representative of three independent experiments. Data are presented as mean±SD. ***P*<0.01, NS not significant.

Fig. S3. Mek1 and Mek2 regulate inflammation and proliferation of MCs under high-glucose conditions. (A)Twenty-four hours after transfection of Mek1 or Mek2 siRNA (50 nM) in H-MC, the Mek1 and Mek2 knockdown efficiencies were detected by qRT-PCR; the knockdown effect of siMek1 No.1 was the best relative to siMek1 No.2 and No.3, and the knockdown effect of siMek2 No.2 was the best one among the three siRNAs. (B) Protein levels of key factors in the Mek/Erk signaling pathway were tested by Western blot and quantitative analysis in H-MC after transfection with siMek1 or siMek2 for 48 hours. (C) Forty-eight hours after transfection with Mek1 or Mek2 siRNA in H-MC, the levels of Mcp-1 and Tnf-α were detected by ELISA. (D) Proliferation of MCs was detected by EdU assay and quantitative analysis after transfection with siMek1 or siMek2 for 48 hours. In all panels, the data are representative of three independent experiments. Data are presented as mean±SD. ^*^*P*<0.05, ^**^*P*<0.01, NS not significant.

Fig. S4. Weight, urine microalbumin, and blood glucose of mice. (A) Weight of mice (n=5). (B) Urine microalbumin of mice (n=5). (C) Blood glucose levels of mice (n=5). The data are representative of three independent experiments. Data are presented as mean±SD. ***P*<0.01.

Fig. S1.

**
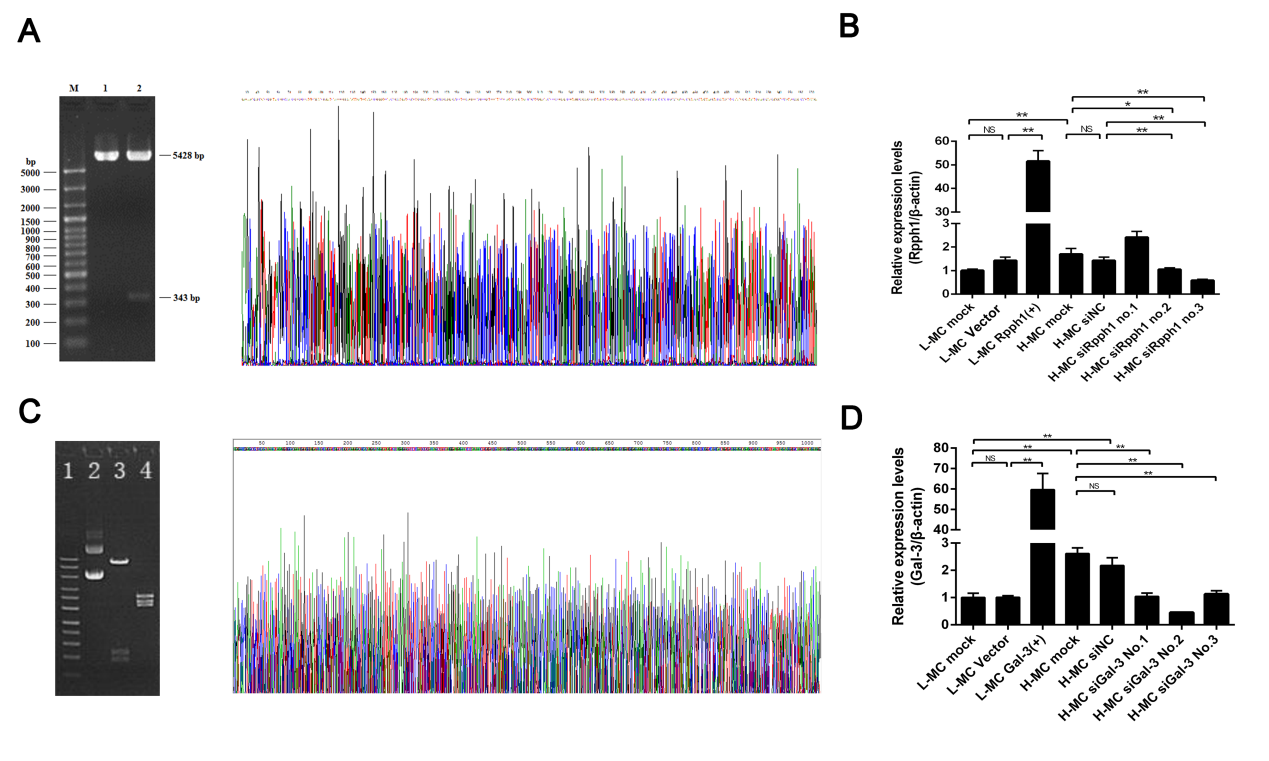
**

Fig. S2.


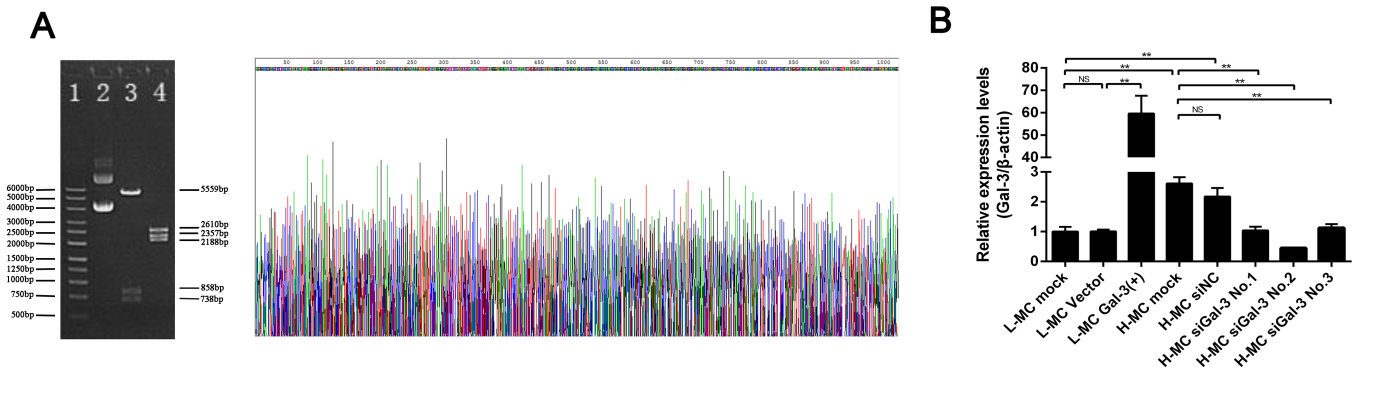


Fig. S3.


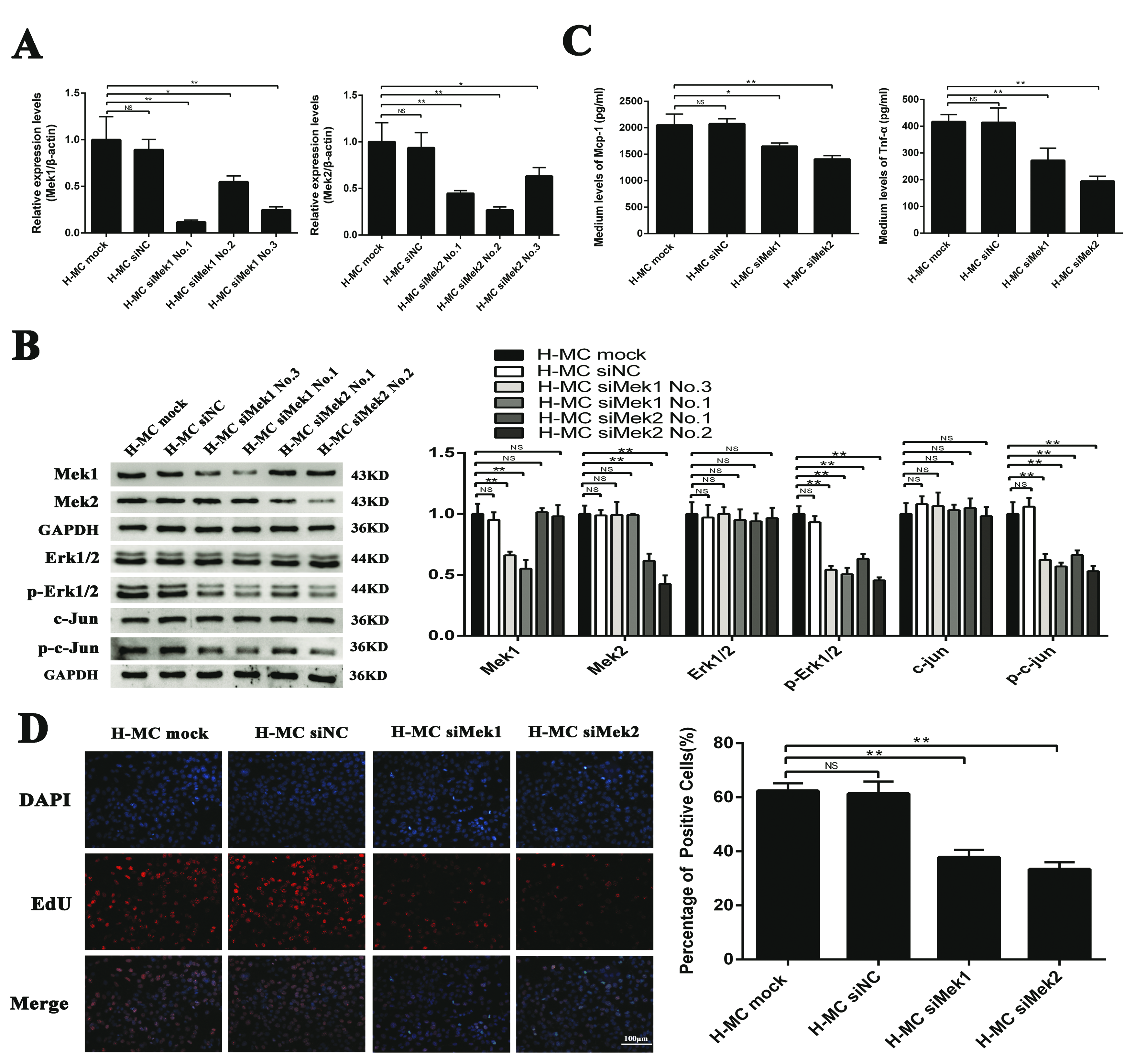


Fig. S4.


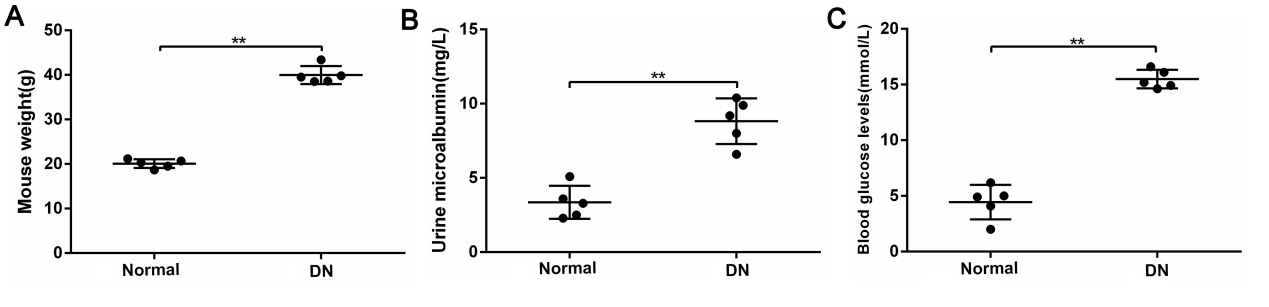

Supplement: Supplementary file 1 — Supplementary data. [file 41419_2019_1765_MOESM1_ESM.docx]
